# Role of Serosal TRPV4-Constituted SOCE Mechanism in Secretagogues-Stimulated Intestinal Epithelial Anion Secretion

**DOI:** 10.3389/fphar.2021.684538

**Published:** 2021-07-14

**Authors:** Yinghui Cui, Fenglan Chu, Kai Yin, Xiongying Chen, Hanxing Wan, Gang Luo, Hui Dong, Feng Xu

**Affiliations:** ^1^ Department of Pediatric Intensive Care Unit, Children’s Hospital of Chongqing Medical University; National Clinical Research Center for Child Health and Disorders; Ministry of Education Key Laboratory of Child Development and Disorders; Chongqing Key Laboratory of Pediatrics, Chongqing, China; ^2^ Department of Gastroenterology, Xinqiao Hospital, Army Medical University, Chongqing, China

**Keywords:** calcium signaling, PGE_2_, 5-HT, SOCE, CRAC, TRPV4, CCH

## Abstract

As little is known about the role of calcium (Ca^2+^) signaling mediating the small intestinal epithelial anion secretion, we aimed to study its regulatory role in secretagogue-stimulated duodenal anion secretion and the underlying molecular mechanisms. Therefore, intestinal anion secretion from native mouse duodenal epithelia was examined with Ussing chambers to monitor PGE_2_-, 5-HT-, and CCh-induced short-circuit currents (*I*_*sc*_). PGE_2_ (10 μM) and 5-HT (10 μM) induced mouse duodenal *I*_*sc*_, markedly attenuated by serosal Ca^2+^-free solution and selective blockers of store-operated Ca^2+^ channels on the serosal side of the duodenum. Furthermore, PGE_2_- and 5-HT-induced duodenal *I*_*sc*_ was also inhibited by ER Ca^2+^ chelator TPEN. However, dantrolene, a selective blocker of ryanodine receptors, inhibited PGE_2_-induced duodenal *I*_*sc*_, while LiCl, an inhibitor of IP_3_ production, inhibited 5-HT-induced *I*_*sc*_. Moreover, duodenal *I*_*sc*_ response to the serosal applications of both PGE_2_ and 5-HT was significantly attenuated in transient receptor potential vanilloid 4 (TRPV4) knockout mice. Finally, mucosal application of carbachol (100 μM) also induced duodenal *I*_*sc*_ via selective activation of muscarinic receptors, which was significantly inhibited in serosal Ca^2+^-free solution but neither in mucosal Ca^2+^-free solution nor by nifedipine. Therefore, the serosal TRPV4-constituted SOCE mechanism is likely universal for the most common and important secretagogues-induced and Ca^2+^-dependent intestinal anion secretion. These findings will enhance our knowledge about gastrointestinal (G.I.) epithelial physiology and the associated G.I. diseases, such as diarrhea and constipation.

## Introduction

Intestinal epithelial ion secretion is a critical physiological process in the human gastrointestinal (G.I.) tract. Since water follows ion movement across osmotic gradients, which is primarily generated by chloride ion (Cl^−^) and bicarbonate (HCO_3_^−^) secretion (Barrett and Keely, 2000; Kiela and Ghishan, 2009), the clarification of these processes is essential to delineate the pathophysiology of various diarrheal diseases. The Cl^−^ and HCO_3_^−^ secretions are under the control of several secretagogues in the G.I. system. Like prostaglandin E_2_ (PGE_2_), is a potent chloride secretagogue likely to be active under physiological and pathophysiological circumstances (Weymer et al., 1985; Rajagopal et al., 2014); meantime, PGE_2_ stimulates duodenal bicarbonate secretion to protect the mucosal epithelium against acid-induced injury in various species (Takeuchi and Amagase, 2018). Furthermore, 5-hydroxytryptamine (5-HT) is also an essential secretagogue of Cl^−^ and HCO_3_^−^ secretion, and it is released by enterochromaffin (E.C.) cells situated in the intestine epithelium (Brown, 1995). Besides, acetylcholine (ACh) is a primary neurotransmitter in activating intestinal anion secretion.

These secretagogues described above mediate epithelial ion transports via three major second messengers: cAMP, cGMP, and Ca^2+^ (Murek et al., 2010). Among these messengers, the physiological roles and molecular mechanisms of cAMP- and cGMP-dependent regulation of intestinal ion transports have been well elucidated (Rao et al., 1984; Tuo et al., 2009), while those mediated via calcium signaling remain relatively poorly understood.

It is commonly believed that in non-excitable cells, secretagogues evoke calcium signaling through two necessary processes: the release of Ca^2+^ from intracellular stores, then an enhanced extracellular Ca^2+^ entry (Putney, 2007), which was called capacitative or store-operated Ca^2+^ channels (SOCs) classically. The intracellular store in the endoplasmic reticulum (E.R.) from which Ca^2+^ is released in two main ways, which is via the ryanodine receptor (RyR) or the inositol trisphosphate receptor (IP_3_R) (MacMillan et al., 2005). These Ca^2+^ release-activated Ca^2+^ channels (CRAC) were first described in mast cells and Jurkat lymphocytes (Hoth and Penner, 1992; Hoth and Penner, 1993). However, detailed underlying mechanisms that secretagogues mediated cytosolic Ca^2+^ signaling in duodenal anion secretion still need to elucidate (Xie et al., 2014). In addition, while molecular components of SOCE are well defined in immune cells, their molecular identification is still elusive in intestinal epithelial cells.

We previously demonstrated that carbachol (CCh), a stable chemical analog of neurotransmitter ACh, triggered IP_3_R/ER Ca^2+^ release, but caffeine triggered RyR/ER Ca^2+^ release, both of which stimulated serosal store-operated Ca^2+^ entry (SOCE) mechanism and eventually induced Ca^2+^-dependent duodenal anion secretion (Yang et al., 2018; Zhang et al., 2019; Zhang et al., 2021) However, it is currently unclear: 1) whether [Ca^2+^]_cyt_ is also a critical cell signaling for other most common and important secretagogues, such as PGE_2_ and 5-HT; 2) if serosal SOCE is a universal mechanism for Ca^2+^-dependent duodenal anion secretion; 3) if so, what molecular components of the SOCE are involved in this process; and 4) if CCh evokes a Ca^2+^-dependent anion secretion when applied from the mucosal side of the duodenum, although it is well known to stimulate it from the serosal side. Therefore, we aimed to investigate these important issues using native duodenal epithelial tissues in mice as a follow-up study.

## Materials and Methods

### Animals and Cells

All experiments were adopted with adult male Harlan C-57BL/6 mice (6–8 weeks old; 18–22 g; Chongqing Tengxin Biotechnology Co. Ltd., Chongqing, China) and transient receptor potential vanilloid 4 (TPPV4) deficient (TRPV4 KO) mice which generated from C-57BL/6 mice (6–12 weeks old; 20–25 g; Cyagen bioscience, China). Animal care and experiments conformed with the guidance of the Animal Ethical Committee of the University and were approved by the University Committee on Investigations Involving Animal Subjects. According to the ARRIVE guidelines (Kilkenny et al., 2010), the mice were bred and housed in a standard animal care room at an ambient temperature of 20°C and air humidity of 50–55% on a 12 h: 12 h light-dark cycle with free access to water and food pellets until the time of experiments. Before each experiment, mice’s food and water were deprived for at least 1 h. Mice were sacrificed by cervical dislocation under narcosis with 100% CO_2_. Animals were assigned randomly to different experimental groups of all studies. Data collection and evaluation of all experiments performed blindly, and the experimenters were unaware of group treatments.

IEC-6, a small intestinal epithelial cell line of rat origin (Thomas and Oates, 2002), was obtained from the American Type Culture Collection (ATCC, Rockville, MD, United States) and routinely cultured in fresh Dulbecco’s modified eagle’s medium (DMEM) supplemented with 10% Fetal bovine serum (FBS), glutamine and penicillin/streptomycin every 2 days. After the cells had grown well for experiments, they were replated onto 12 mm round coverslips (Warner Instruments Inc., Hamden, CT, United States) and incubated for at least 24 h before use for [Ca^2+^]_cyt_ measurement.

### Solutions

Solutions to the mucosal side in the Ussing chamber experiments contained the following: 115 mM NaCl, 25 mM sodium-D-gluconate, 5.2 mM potassium-D-gluconate, 1.2 mM CaCl_2_, 1.2 mM MgCl_2_, and 10 mM D-mannitol at pH 7.4 when gassed with Oxygen (100% O_2_) at 37°C. The serosal solution contained the following: 115 mM NaCl, 25 mM NaHCO_3_, 2.2 mM K_2_HPO_4_, 1.2 mM CaCl_2_, 1.2 mM MgCl_2_, 0.8 mM KH_2_PO_4_, 10 mM D-glucose, and this solution was gassed with carbogen (5% CO_2_ and 95% O_2_, v/v) at 37°C and had a pH 7.4. For the Ca^2+^-free experiments, Ca^2+^ was omitted, and EGTA (0.5 mM) was added in both mucosal and serosal solutions to prevent potential Ca^2+^ contamination. The physiological salt solution (PSS) used in digital Ca^2+^ measurement contained the following: 140 mM NaCl, 5 mM KCl, 2 mM CaCl2, 10 mM HEPES, and 10 mM glucose (pH 7.4). For the Ca^2+^-free PSS solution, Ca^2+^ was omitted, but 0.5-mM EGTA was added. The osmolalities for all solutions were 300 mOsmol kg^−1^ of H_2_O.

### Tissue Preparations

Following euthanasia, the mice’s abdomen was opened by a midline incision. Next, we dissected the proximal duodenum 4 cm from the pylorus carefully and immediately but not pulled to avoid damaging the epithelium. Afterward, the duodenum section was incubated in ice-cold iso-osmolar mannitol (300 mM) and indomethacin (1μM) solution 10 min before seromuscular stripping to inhibit possible endogenous PGE_2_, which is resulting from mucosal injury during experiments, to avoid affecting the basal *I*_*sc*_. Finally, the section is opened longitudinally, with the mesenteric attachment remnant, seromusculature stripped, and divided into four segments. The segment, which is likely to undergo less excision damage, will be situated in the chambers’ aperture (window area, 0.1 cm^2^).

### Ussing Chamber Experiments

Segments were fixed in a modified Ussing chamber bathed with a volume of 3 ml on each side of the mucosa preparation at 37°C and short-circuited by a computer-controlled voltage-clamp device (Voltage-Current Clamp, VCC MC6; Physiologic Instruments, San Diego, CA, United States) under continuous short-circuited conditions. An automatic voltage-clamp measured the transepithelial short-circuit currents (*I*_*sc*_), while μA was used for the original recordings, and μA cm^−2^ was used for summary data. After 10–15 min of measurements for basal parameters, various agonists or antagonists were added to one side or both sides for 10–20 min, followed by PGE_2_, 5-HT, and carbachol.

### Calcium Image Experiments

[Ca^2+^]_cyt_ measurement experiments were performed as previously described (Zhang et al., 2019). ICE-6 Cells grown on coverslips were loaded with 5 μM Fura-2/AM in PSS, described above, at room temperature (22–25°C) for 50 min and then washed for 30 min. After that, the coverslips with epithelial cells were mounted in a perfusion chamber on a Nikon microscope stage (Nikon Corp., Tokyo, Japan). The ratio of Fura-2/AM fluorescence with excitation at 340 or 380 nm (F340/380) was followed over time and captured using an intensified charge-coupled device camera (ICCD200) and a MetaFluor imaging system (Universal Imaging Corp., Downingtown, PA, United States).

### Materials

Prostaglandin E_2_, Serotonin hydrochloride(5-HT), SKF-96365, TPEN (N, N, N′, N′- tetrakis (2-pyridylmethyl) ethylenediamine), GSK-7975A, ouabain, HC067047, and GSK1016790A were purchased from MedChemExpress (MCE; Monmouth Junction, NJ, United States). Sigma (Saint Louis, MO, United States) supplied carbamylcholine chloride (CCh), nifedipine, gadolinium chloride, and cyclopiazonic acid (CPA). Tocris Bioscience (Ellisville, MO, United States) supplied 2-Aminoethoxydiphenyl borate (2-APB), while APExBIO Technology LLC (Houston, TX, United States) provided dantrolene. Fura-2 was purchased from Invitrogen (UT, United States), meantime DMEM and FBS were obtained from Hyclone (Logan, UT, United States). Trypsin and penicillin/streptomycin were purchased from Gibco (CA, United States). The other chemicals were obtained from BBI Life Science (Shanghai, China).

### Data and Statistical Analysis

Data and statistical analysis yield to the recommendations of Frontiers in Pharmacology. All results are given as means ± standard error of the mean number (*n*) of investigated tissues. Net peak of duodenal *I*_*sc*_ refers to drug-stimulated maximal peak minus basal level. The statistical significance of differences in experimental groups’ means was determined by using Student’s unpaired, two-tailed t-test or one-way ANOVA followed by Dunnett’s post-test. Post hoc tests were run if F achieved *p* < 0.05 (GraphPad Prism 8.0), and there was no significant variance in inhomogeneity. A probability *P-value* <0.05 was considered statistically significant.

## Results

### Prostaglandin E_2_ Induced Ca^2+^-Dependent Epithelial Anion Secretion in Duodenum

**FIGURE 1:**
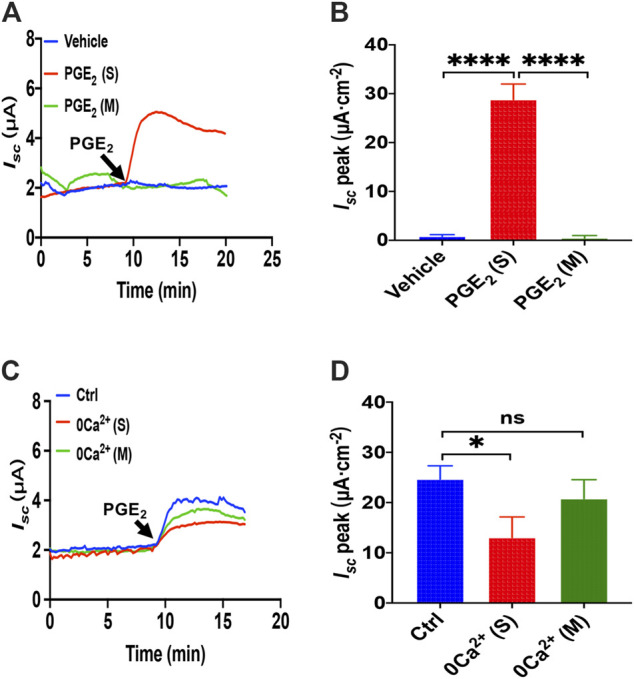
PGE_2_ induced Ca^2+^-dependent small intestine epithelial anion secretion. **(A)** Time courses of PGE_2_ (10 μM) -evoked *I*_*sc*_ or vehicle (DMSO) when applied to the serosal (s) or mucosal (m) side of mice duodenal mucosal tissues (*n* ≥ 6). **(B)** When added to the serosal or mucosal side, vehicle or PGE_2_-stimulated *I*_*sc*_ peak (*n* = 6). **(C)** Time courses of PGE_2_-evoked *I*_*sc*_ after extracellular Ca^2+^ omission (0 Ca^2+^) from each side in duodenal mucosal tissues. **(D)** PGE_2_-stimulated *I*_*sc*_ peak after Ca^2+^ omission from each side (*n* ≥ 6). Ctrl represents as the control in which normal extracellular Ca^2+^ was on both sides. Results are presented as mean ± SE. *p < 0.05, ****p < 0.0001, significantly different from the corresponding control by one-way ANOVA followed by Dunnett’s post-test.

Since PGE_2_ is one of the most common and important secretagogues, we conducted Ussing chamber experiments to test its effect on Ca^2+^-dependent duodenal epithelial anion transports. Because the duodenal epithelium is polarized, with the mucosal side and the serosal side, we tested which side was acted by PGE_2_. The addition of PGE_2_ (10 μM) in the serosal induced a transient high *I*_*sc*_ peak with a sustained phase following (Figure 1A). However, PGE_2_ and vehicle (DMSO) mucosal application did not affect the duodenal *I*_*sc*_ (Figures 1A,B). Therefore, PGE_2_ acts on the serosal side of the duodenum exclusively.

To examine whether extracellular Ca^2+^ is vital for PGE_2_-evoked anion secretion, we omitted extracellular Ca^2+^ on the serosal or mucosal sides of the Ussing chamber. We found that calcium omission of the serosal side weakened the PGE_2_-evoked *I*_*sc*_ peak but not the mucosal side (Figures 1A,B). Therefore, the presence of Ca^2+^ in the serosal side is critical for PGE_2_-induced duodenal *I*_*sc*_.

### Prostaglandin E_2_ Induced Ca^2+^-Dependent Ion Secretion by Serosal Store-Operated Ca^2+^ Entry Mechanism

**FIGURE 2:**
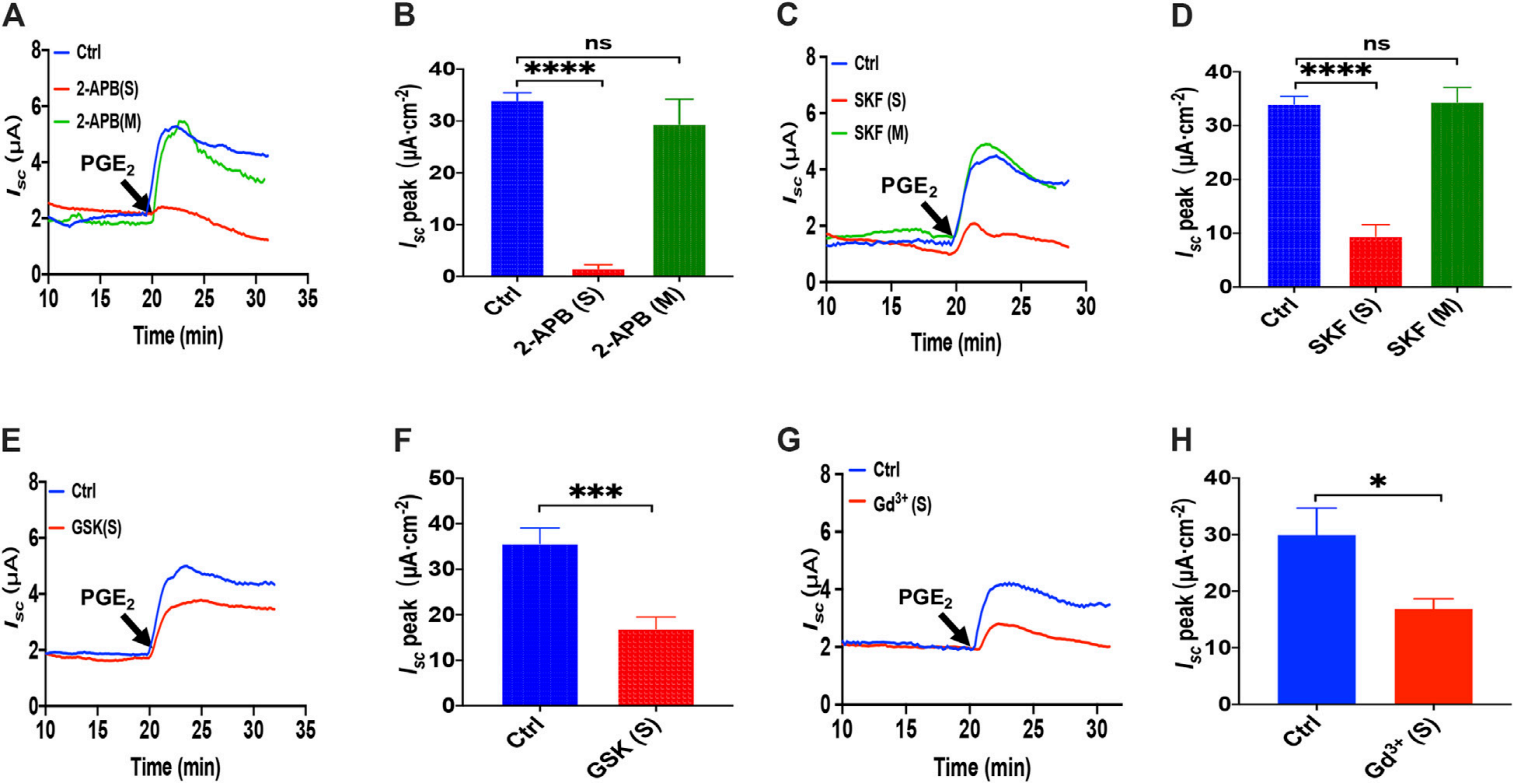
PGE_2_ induced Ca^2+^-dependent ion secretion by serosal SOCE mechanism in mice duodenum. **(A)** Time course of PGE_2_ (10 μM) -evoked *I*_*sc*_ after mucosal or serosal application of 2-aminoethoxydiphenyl borate (2-APB, 100 μM, *n* = 6). **(B)** PGE_2_-evoked *I*_*sc*_ peak after 2-APB was added to the serosal or mucosal side. **(C)** Time course of PGE_2_-evoked *I*_*sc*_ after mucosal or serosal application of SKF-96365 (SKF, 30 μM, *n* = 6). **(D)** PGE_2_-evoked *I*_*sc*_ peak after SKF-96365 was added to the serosal or mucosal side. **(E)** Time course of PGE_2_-evoked duodenal *I*_*sc*_ after serosal application of GSK-7975A (GSK, 100 μM, *n* = 6). **(F)** PGE_2_-evoked duodenal *I*_*sc*_ peak after serosal application of GSK-7975A. Ctrl represents the control without drug treatment. **(G)** Representative of the time course of PGE_2_-stimulated duodenal *I*_*sc*_ after serosal addition of GdCl_3_ (Gd^3+^, 30 μM, *n* = 6). **(F)** Summary of the effect of GdCl_3_ on PGE_2_-stimulated duodenal *I*_*sc*_ peak after serosal addition. Ctrl represents the control without drug treatment. Results are presented as mean ± SE. NS, no significant differences, *p < 0.05, ***p < 0.001, ****p < 0.0001 vs. corresponding control by Student’s unpaired, two-tailed t-test or one-way ANOVA followed Dunnett’s post-test.

To examine if the SOCE mechanism was involved in PGE_2_-mediated anion transports, we adapted four inhibitors with different chemical structures to block SOCE. Considering 2-APB is a SOCE and an inconsistent IP_3_R inhibitor (Bootman et al., 2002), we first applied 2-APB to test. We found that 2-APB (100 μM) had no effect on PGE_2_-stimulated duodenal *I*_*sc*_ after mucosal addition while adding in the serosal side significantly attenuated the duodenal *I*_*sc*_ (Figures 2A,B). Like 2-APB, SKF-96365, a selective SOCE blocker added in the serosal side but not the mucosal side, also significantly suppressed the duodenal *I*_*sc*_ (Figures 2C,D).

Since GSK-7975A is a specific blocker of the CRAC channel (Molnar et al., 2016), we tested if the CRAC channel is SOCE in the duodenal epithelium utilizing it. As shown in Figures 2E,F, GSK-7975A (100 μM) markedly reduced PGE_2_-stimulated duodenal *I*_*sc*_. Furthermore, considering Gd^3+^ has been the most widely employed tool for blocking SOCE and CRAC/Orai channel (Bird et al., 2008; Sataloff et al., 2017), we added GdCl_3_ (30 μM) in the serosal side significantly reduced PGE_2_-stimulated duodenal *I*_*sc*_ (Figures 2G,H). Therefore, PGE_2_ induced Ca^2+^-dependent ion secretion by acting on the serosal SOCE mechanism and probably CRAC channels in the duodenal epithelium.

### ER Ca^2+^ Store and Ryanodine Receptors in PGE_2_-Induced Intestinal Ion Transports

**FIGURE 3:**
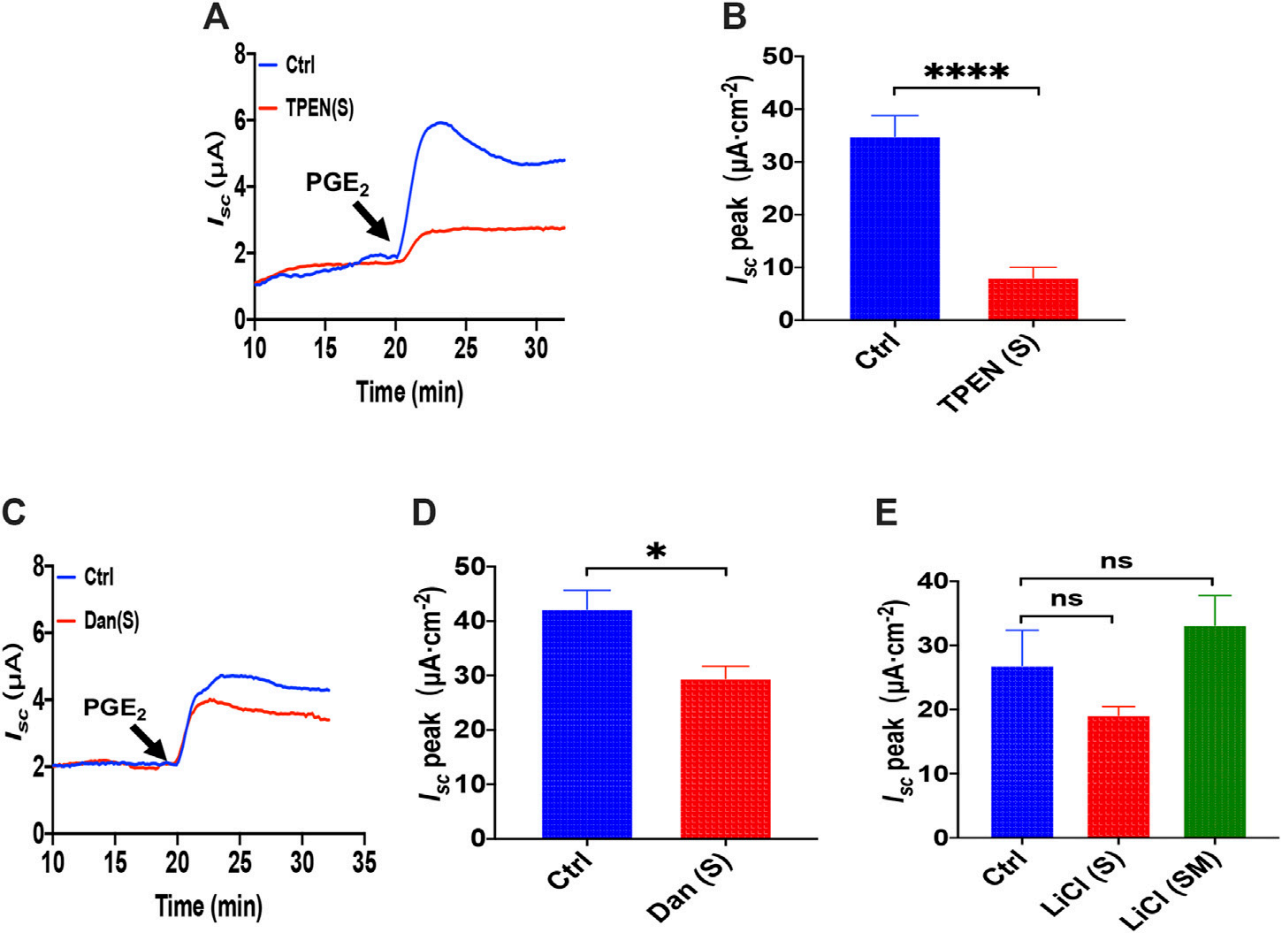
The ER Ca^2+^ store and ryanodine receptors in PGE_2_ -induced duodenal ion transports. **(A,B)** PGE_2_ (10 μM) -evoked *I*_*sc*_ after serosal (s) application of TPEN (1 mM, *n* = 6). **(C,D)** PGE_2_-evoked *I*_*sc*_ after dantrolene (Dan, 300 μM, *n* ≥ 6) was added to the serosal (s). **(E)** PGE_2_-evoked *I*_*sc*_ after LiCl (30 mM, n ≥ 6) was added to the serosal side or both sides (m + s). Ctrl represents the control without drug treatment. Results are presented as mean ± SE. NS, no significant differences, *p < 0.05, ****p < 0.0001 vs. corresponding control by Student’s unpaired, two-tailed t-test or one-way ANOVA followed by Dunnett’s post-test.

As is know that N, N, N′, N′-tetrakis (2-pyridylmethyl) ethylenediamine (TPEN) can rapidly and reversibly chelate Ca^2+^ within E.R. stores without influencing [Ca^2+^]_cyt_ for its low affinity with Ca^2+^ (Caroppo et al., 2003), which we applied to investigate further the role of ER Ca^2+^ store in PGE_2_-induced duodenal *I*_*sc*_. As shown in Figures 3A,B, serosal addition of TPEN (1 mM) significantly suppressed PGE_2_-evoked *I*_*sc*_. Considering ryanodine receptors (RyR) can mediate Ca^2+^ release from E.R., serosal addition of dantrolene (300 μM), a selective RyR antagonist, markedly inhibited PGE_2_-induced *I*_*sc*_ (Figures 3C,D), suggesting E.R. Ca^2+^ store release dominantly by the serosal side. As the inositol 1,4,5-triphosphate (IP_3_) also leads to E.R. intracellular Ca^2+^release through E.R. membrane by IP_3_ receptors (Lindqvist et al., 1998), we used LiCl (30 mM) that inhibits IP_3_ production, but LiCl added either on the serosal side or both sides of the tissues did not significantly alter PGE_2_-induced *I*_*sc*_ (Figure 3E). These findings suggest that PGE_2_ acts via RyR/Ca^2+^ rather than IP_3_/Ca^2+^ in E.R. to induce duodenal epithelial anion transports.

### Prostaglandin E_2_ Induced Duodenal Ion Secretion by Serosal Transient Receptor Potential Vanilloid 4 Channels and Na^+^/K^+^ ATPase

**FIGURE 4:**
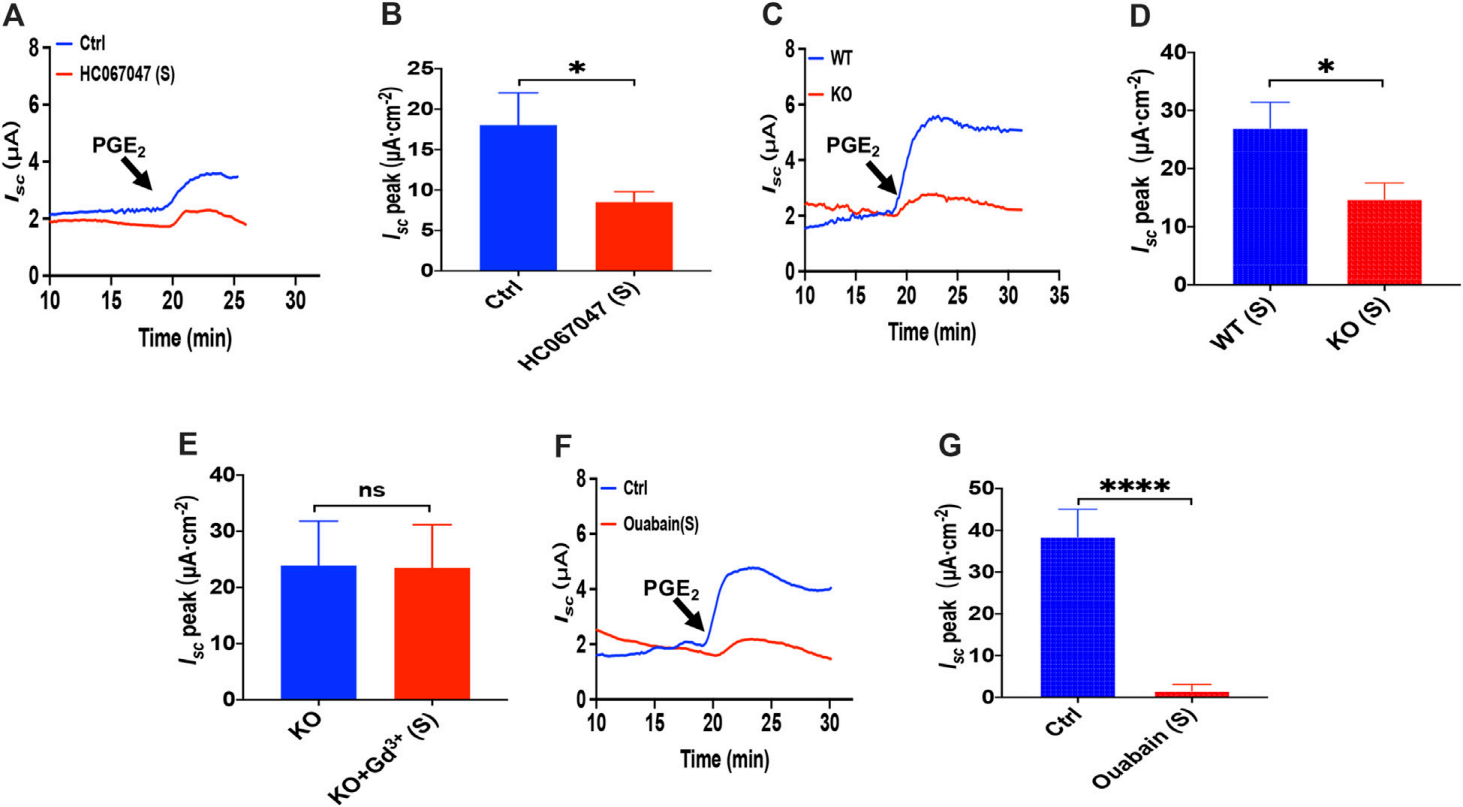
PGE_2_ induced duodenal ion secretion by serosal TRPV4 channels and NKA. **(A,B)** on PGE_2_-evoked *I*_*sc*_ after HC067047 (30 μM, *n* = 6) added in serosal (s) side. **(C,D)** PGE_2_ (10 μM) -evoked duodenal *I*_*sc*_ in wild-type (W.T.) and TRPV4 knockout (K.O.) mice (*n* = 6). **(E)** PGE_2_-evoked *I*_*sc*_ after GdCl_3_ (Gd^3+^, 30 μM, *n* ≥ 6) was added to the serosal (s) side of the TRPV4 KO mice duodenum. **(F,G)** PGE_2_-stimulated *I*_*sc*_ after Ouabain (1 mM, *n* = 7) was added to the serosal (s) side. Ctrl represents the control without drug treatment. Results are presented as mean ± SE. *p < 0.05, ****p < 0.0001 significantly different from the corresponding control by Student’s unpaired, two-tailed t-test.

Since that 2-APB, SKF-96365 and GSK-7975A also act on the transient receptor potential V(TRPV) family and that TRPV4 channels are expressed in the G.I. tract (Blackshaw et al., 2010), we tested if TRPV4 channel may represent the molecular constituents of CRAC channels in the process of the PGE_2_-stimulated duodenal *I*_*sc*_. We all know that GSK1016790A, a highly selective agonist of TRPV4, can activate TRPV4 in diverse cells (Baratchi et al., 2019). However, unlike the effect in cultured cells detected in the ussing chamber at the tissue level, GSK1016790A alone had no effect on the basal *I*_*sc*_ (Supplementary Figure S1). Hence we first chose the application of HC067047, a potent and selective TRPV4 antagonist (Xia et al., 2013), to block TRPV4 Channels. As shown in Figures 4A,B, serosal addition of HC067047 (30 μM) suppressed PGE_2_-stimulated duodenal *I*_*sc*_ *.* Secondly, we compared PGE_2_-stimulated duodenal *I*_*sc*_ between wild-type and TRPV4 KO mice. Duodenal *I*_*sc*_ induced by serosal addition of PGE_2_ was significantly attenuated in TRPV4 KO mice (Figures 4C,D), while HC067047 (30 μM) did not affect PGE_2_ evoked *I*_*sc*_ of TRPV4 KO mice to exclude non-specific effects other than TRPV4 inhibition (Supplementary Figure S2). Thirdly, to further verify TRPV4 involved in the molecular composition of SOCE, we used SOCE and CRAC blocker, Gd^3+^, to test whether it works on TRPV4 knockout mice. GdCl_3_ (30 μM) serosal addition did not affect the PGE_2_-stimulated duodenal *I*_*sc*_ of TRPV4 knockout mice. Above all, our findings are suggesting that TRPV4 is the molecular constituent of CRAC channels.

Since Cl^−^ movement across the epithelial cells is facilitated by Na^+^/K^+^ ATPase (NKA), we tested if it is involved in the process of PGE_2_-stimulated duodenal *I*_*sc*_. Because NKA is exclusively expressed at the serosal side in the intestinal epithelium (Hammerton et al., 1991; Thorsen et al., 2014), addition of 1 mM ouabain, a selective NKA inhibitor, to the serosal side completely abolished PGE_2_-induced duodenum *I*_*sc*_ (Figures 4F,G), indicating that NKA participates in PGE_2_-mediated duodenal secretion.

### 5-HT Induced Ca^2+^-Dependent Duodenal Epithelial Ion Transports

**FIGURE 5:**
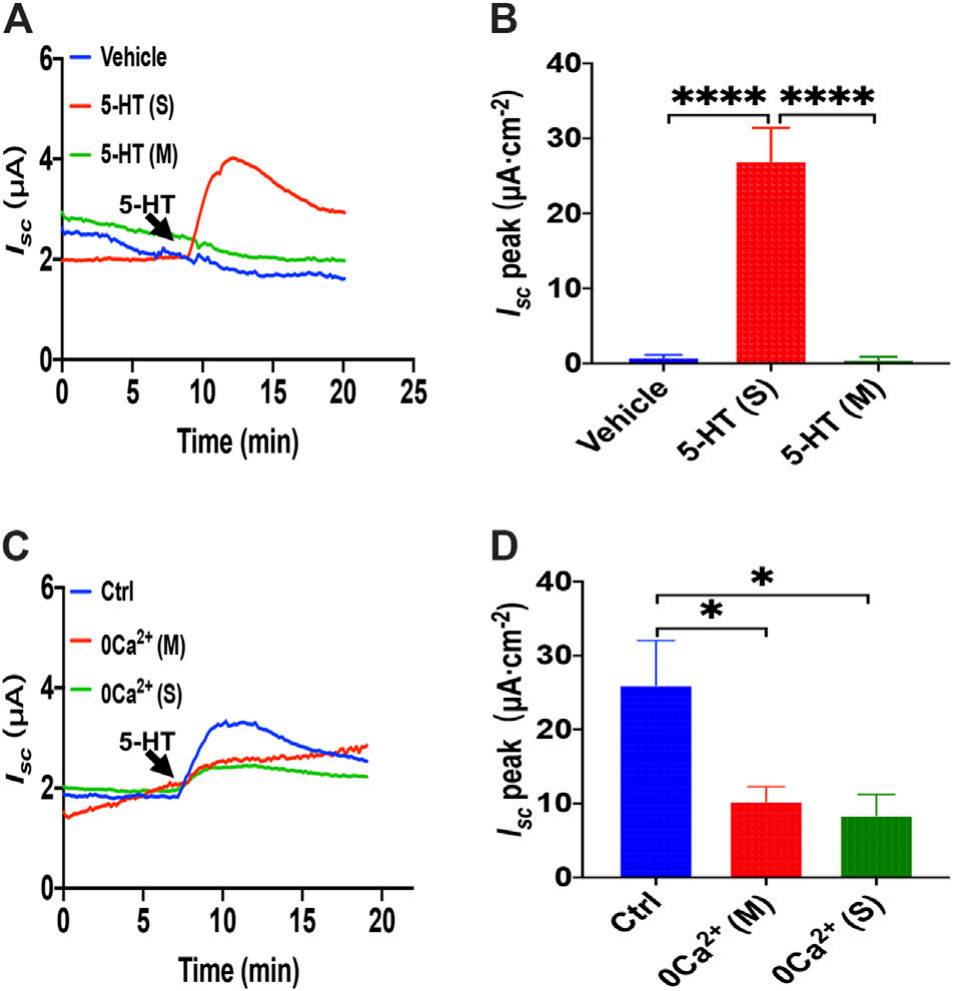
5-HT induced Ca^2+^-dependent duodenal epithelial ion transports. **(A)** Time courses of 5-HT (10 μM) -evoked *I*_*sc*_ or vehicle (DMSO) when added to serosal (s) or mucosal side. **(B)** When added to the serosal or mucosal side, vehicle or 5-HT-evoked *I*_*sc*_ peak (*n* = 6). **(C)** Time courses of 5-HT-evoked *I*_*sc*_ after extracellular Ca^2+^ omission (0 Ca^2+^) from each side in duodenal mucosal tissues. **(D)** 5-HT-evoked *I*_*sc*_ peak after Ca^2+^ omission from each side (s or m, *n* = 6). Ctrl represents as the control in which normal extracellular Ca^2+^ was on both sides. Results are presented mean ± SE. *p < 0.05, ****p < 0.0001 significantly different from the corresponding control by one-way ANOVA followed by Dunnett’s post-test.

When another important secretagogue, 5-HT (10 μM), was added to the serosal side, duodenal *I*_*sc*_ increased, peaking within 2 min, and then sustained for more than 10 min (Figures 5A,B). However, mucosal application of 5-HT or vehicle (DMSO) did not alter basal *I*_*sc*_. Therefore, to test if Ca^2+^ is involved in the process of 5-HT-stimulated duodenal ion transports, we omitted extracellular Ca^2+^ of each side, and then we found that 5-HT- evoked *I*_*sc*_ was markedly suppressed in either side of the duodenal tissues (Figures 5C,D). Therefore, 5-HT-stimulated duodenal ion transports are strongly Ca^2+^-dependent.

### 5-HT Induced Ca^2+^-Dependent Intestinal Ion Secretion by Serosal Store-Operated Ca^2+^ Entry Mechanism

**FIGURE 6:**
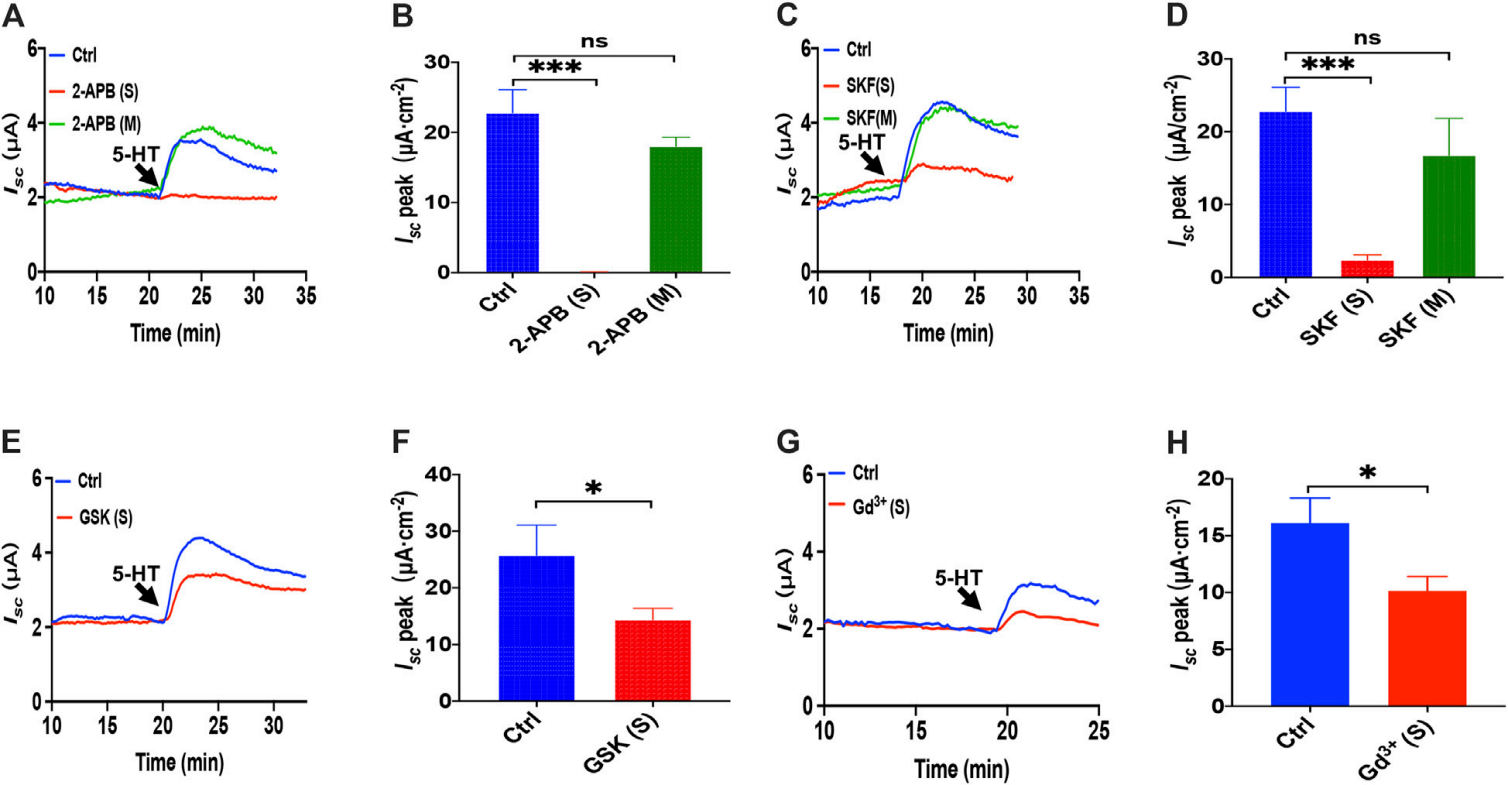
5-HT induced Ca^2+^-dependent intestinal ion secretion by serosal SOCE mechanism in mice duodenum. **(A)** Time course of 5-HT-evoked *I*_*sc*_ after 2-aminoethoxydiphenyl borate (2-APB, 100 μM, *n* = 6) added to the serosal or mucosal side. **(B)** 5-HT-stimulated murine duodenal *I*_*sc*_ peak after 2-APB was added to the serosal or mucosal side. **(C)** Time course of 5-HT-evoked *I*_*sc*_ after SKF-96365 (SKF, 30 μM, *n* = 6) added to the serosal or mucosal side. **(D)** 5-HT-evoked duodenal *I*_*sc*_ peak after SKF-96365 was added to the serosal or mucosal side. **(E)** Time course of 5-HT-evoked *I*_*sc*_ after GSK-7975A (GSK, 100 μM, *n* = 6) added to the serosal side. **(F)** 5-HT-evoked *I*_*sc*_ peak after GSK-7975A was added to the serosal side. **(G-H)** 5-HT-evoked *I*_*sc*_ after GdCl_3_ (Gd^3+^, 30 μM, *n* = 6) added to the side. Ctrl represents the control without drug treatment. Results are presented as mean ± SE. NS, no significant differences, *p < 0.05, ***p < 0.001 vs. corresponding control by Student's unpaired, two-tailed t-test or one-way ANOVA followed Dunnett’s post-test.

Because it is still elusive for the mechanisms underlying 5-HT induced Ca^2+^-dependent duodenal ion secretion, we examined if the above mechanisms similar to PGE_2_ are involved. First, we utilized three antagonists of SOCE. 2-APB (100 μM) application to the serosal side significantly suppressed 5-HT-evoked *I*_*sc*_, but the mucosal side application did not affect *I*_*sc*_ (Figures 6A,B). SKF-96365 (30 μM) markedly decreased the *I*_*sc*_ peak from serosal side application but not from the mucosal side of the duodenum (Figures 6C,D). Hence, 5-HT also mediates SOCE mechanisms that act entirely on the duodenal serosal side, consistently with our findings with PGE_2_ described above.

We further identified if the CRAC channel is 5-HT-mediated SOCE in the duodenal epithelium by utilizing GSK-7975A and Gd^3+^. When GSK-7975A (100 μM) was added to the serosal side, 5-HT-stimulated duodenal *I*_*sc*_ *is* markedly inhibited in duodenal tissue (Figures 6E,F). Furthermore, GdCl_3_ (30 μM) application on the serosal side also significantly reduced 5-HT-stimulated duodenal *I*_*sc*_ (Figures 6G,H). Therefore, 5-HT induced Ca^2+^-dependent intestinal ion secretion by serosal SOCE mechanism and probably CRAC channels.

### 5-HT Induced Duodenal Ion Secretion via Serosal Transient Receptor Potential Vanilloid 4 Channels

**FIGURE 7:**
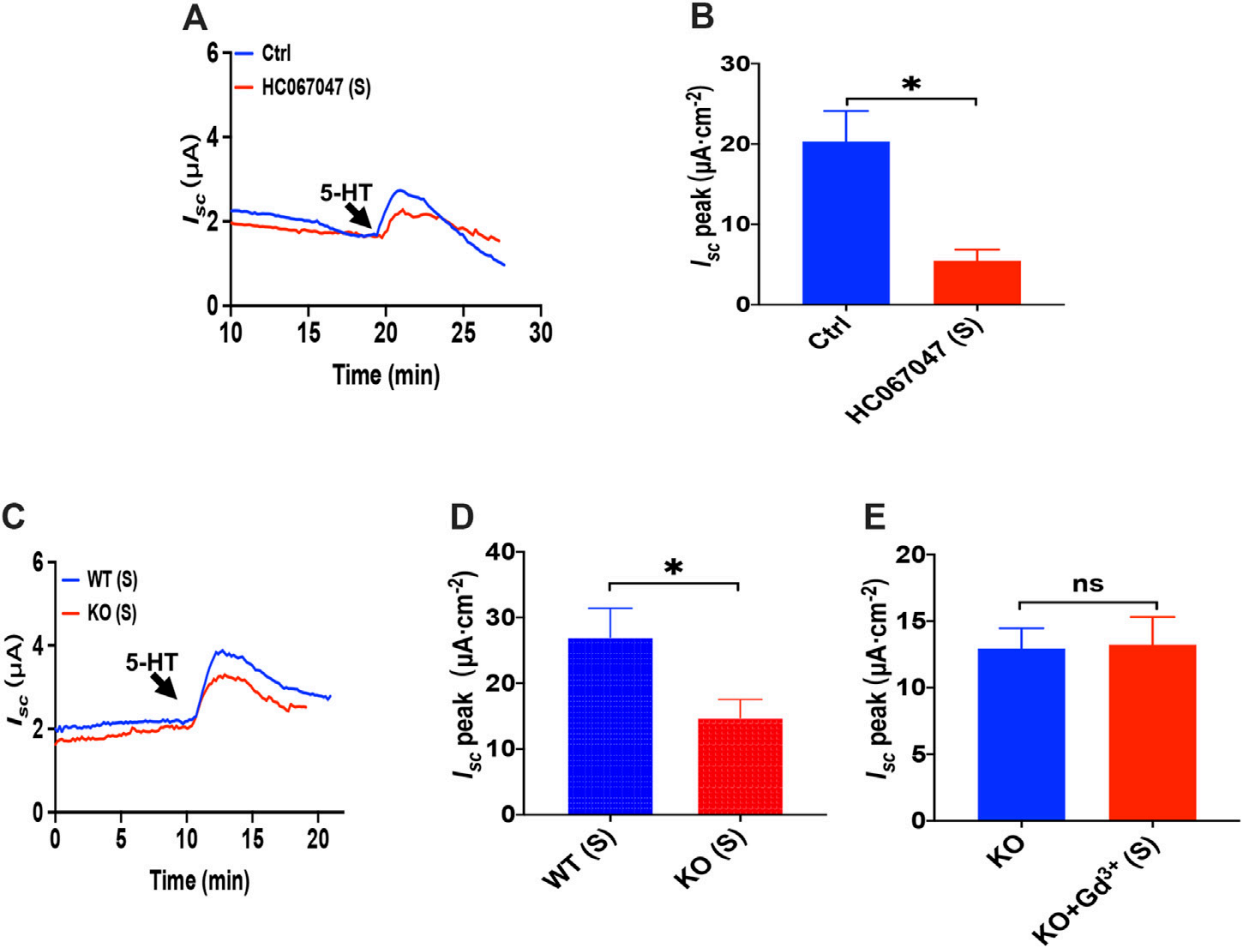
5-HT induced duodenal ion secretion via serosal TRPV4 channels. **(A,B)** 5-HT-evoked *I*_*sc*_ after HC067047 (30 μM, *n* = 6) was added to the serosal (s) side. **(C,D)** 5-HT (10 μM) -evoked *I*_*sc*_ in wild-type (W.T.) or TRPV4 knockout (K.O.) mice (*n* = 6). **(E)** 5-HT-evoked *I*_*sc*_ after GdCl (Gd^3+^, 30 μM, *n* = 6) was added to the serosal (s) side of the TRPV4 KO mice duodenum. Ctrl represents the control without drug treatment. Results are presented as mean ± SE. NS, no significant differences, *p < 0.05 vs. corresponding control by Student’s unpaired, two-tailed t-test.

Like PGE_2_ experiments, we first chose HC067047, a potent and selective TRPV4 antagonist, to block TRPV4 channels. As shown in Figures 7A,B, serosal addition of HC067047 (30 μM) suppressed the 5-HT-stimulated duodenal *I*_*sc*_ *.* Secondly, the duodenal *I*_*sc*_ response to serosal application of 5-HT was significantly attenuated in TRPV4 knockout mice (Figures 7C,D), while HC067047 (30 μM) did not affect PGE_2_ evoked *I*_*sc*_ of TRPV4 KO mice to exclude non-specific effects other than TRPV4 inhibition (Supplementary Figure S3). Thirdly, GdCl_3_ (30 μM) serosal addition did not affect the 5-HT-stimulated duodenal *I*_*sc*_ of TRPV4 knockout mice (Figure 7E). Therefore, the present studies suggest that TRPV4 is the molecular constituent of CRAC channels in the duodenum again.

### The ER Ca^2+^ Store and IP_3_/Ca^2+^ Signaling in 5-HT-Induced Intestinal Ion Transports

**FIGURE 8:**
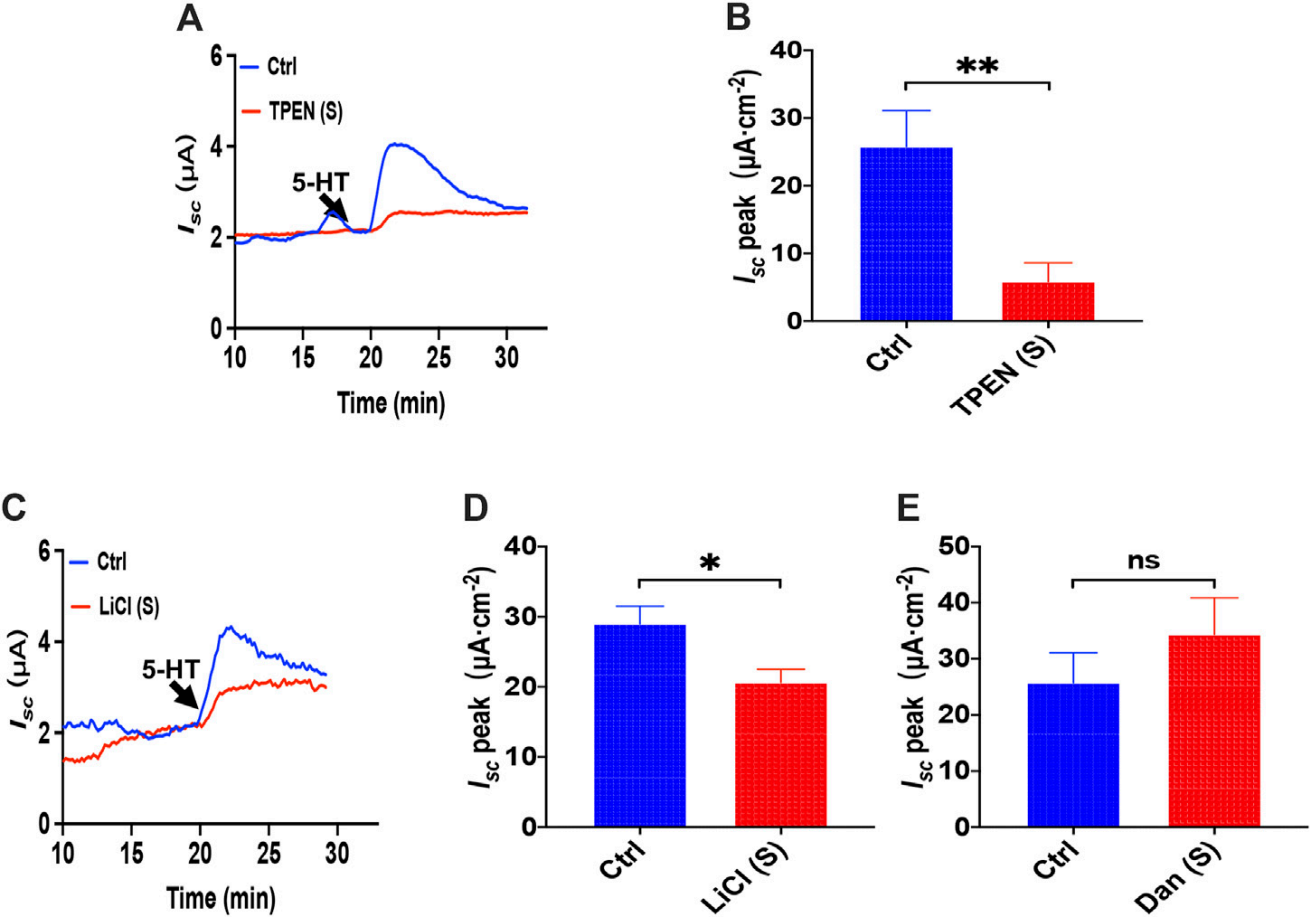
The ER Ca^2+^ store and IP_3_/Ca^2+^ signaling in 5-HT-induced intestinal ion transports. **(A,B)** 5-HT-evoked duodenal *I*_*sc*_ after TPEN (1 mM, *n* = 6) added to serosal (s) side. **(C,D)** 5-HT-evoked *I*_*sc*_ after LiCl (30 mM, *n* = 6) was added to the serosal (s) side. **(E)** 5-HT-evoked *I*_*sc*_ after dantrolene (Dan, 100 μM, *n* = 6) was added to the serosal side. Ctrl represents the control without drug treatment. Results are presented as mean ± SE. NS, no significant differences, *p < 0.05, **p < 0.01 vs. corresponding control by Student’s unpaired, two-tailed t-test.

To investigate how the ER Ca^2+^ store act in 5-HT-evoked *I*_*sc*_, we added TPEN (1 mM) to serosal side of the duodenum and found that TPEN significantly inhibited 5-HT-stimulated *I*_*sc*_ (Figures 8A,B), indicating a vital role of the ER Ca^2+^ store in this course. Then we used LiCl (30 mM) to inhibit IP_3_ production but dantrolene (300 μM) to inhibit RyR. Interestingly, we found LiCl but not dantrolene significantly inhibited 5-HT-induced duodenal *I*_*sc*_ (Figures 8C–E). Therefore, unlike PGE_2_, 5-HT induced duodenal epithelial anion transports via IP_3_/Ca^2+^ rather than RyR/Ca^2+^ in the E.R.

### Luminal Carbachol Induced Ca^2+^-Dependent Duodenal *I*_*sc*_ Through Serosal Ca^2+^ Entry

**FIGURE 9:**
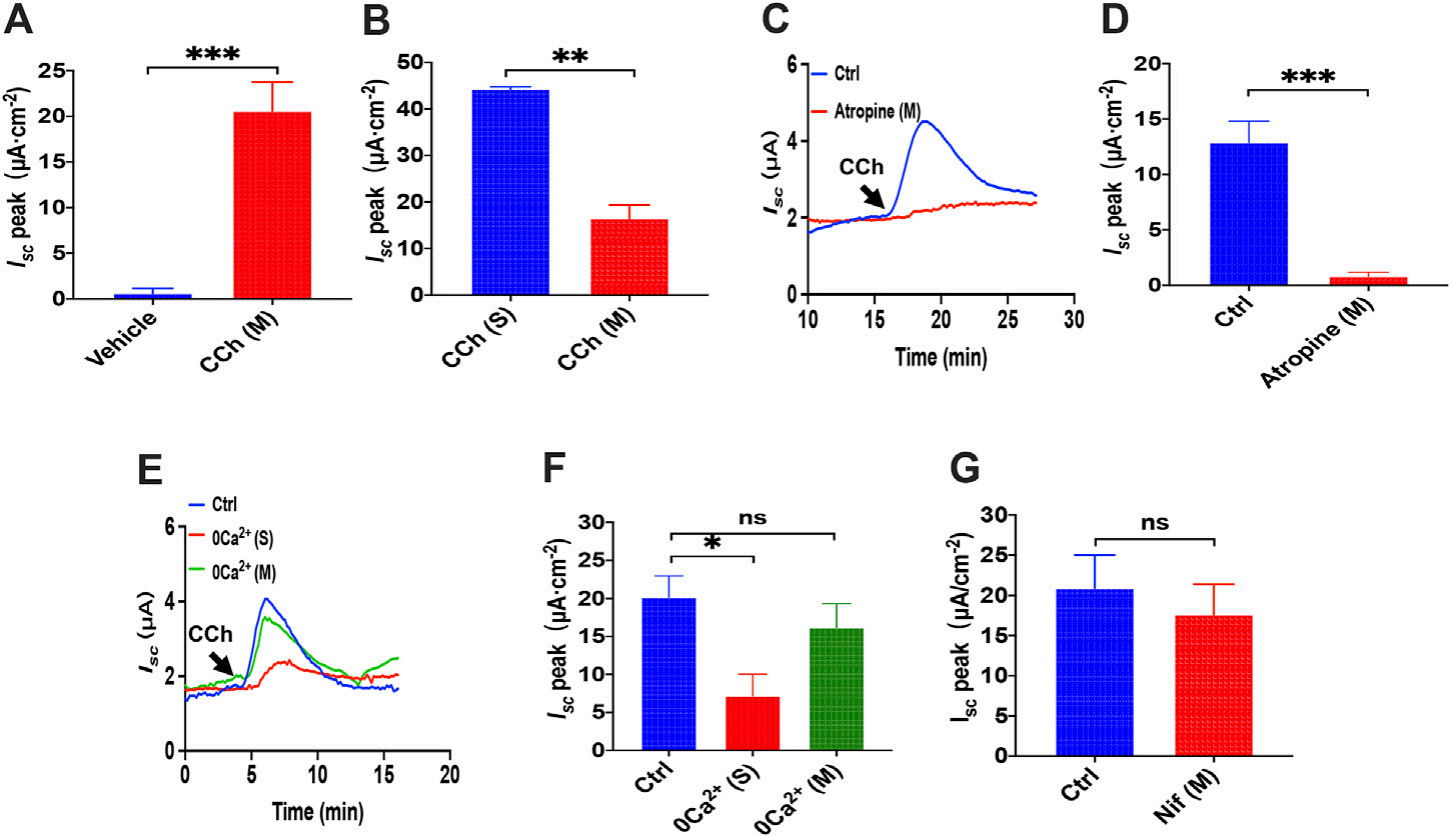
luminal CCh induced Ca^2+^-dependent duodenal *I*_*sc*_ through serosal Ca^2+^ entry. **(A)** Summary data of vehicle (H_2_O) or CCh (100 μM) -stimulated *I*_*sc*_ peak after mucosal application (*n* = 6). **(B)** Comparison between CCh-evoked *I*_*sc*_ peak after mucosal and serosal application. **(C,D)** luminal CCh-induced duodenal *I*_*sc*_ after atropine (10 μM, *n* = 6) added to the mucosal side. Ctrl represents the control without atropine treatment. **(E,F)** luminal CCh-evoked *I*_*sc*_ after extracellular Ca^2+^ omitted from the serosal or mucosal side (*n* = 6). Ctrl represents as the control in which normal extracellular Ca^2+^ was on both sides. **(G)** luminal CCh-evoked duodenal *I*_*sc*_ after mucosal addition of Nifedipine (Nif, 10 μM, *n* = 6). Ctrl represents the control without Nif treatment. Results are presented as mean ± SE. *p < 0.05, **p < 0.01, ***p < 0.001 vs. corresponding control by Student’s unpaired, two-tailed t-test.

We previously demonstrated a critical role of serosal SOCE mechanism mediated by CCh, one of the most common and important secretagogues in Ca^2+^-dependent duodenal ion secretion. (Yang et al., 2018); however, it is not known if luminal addition of CCh can induce duodenal ion transports. Unlike PGE_2_ and 5-HT that act on seroal side of the duodenum exclusively (Figures 1A, 5A), we found that luminal addition of CCh (100 μM) induced a significant duodenal *I*_*sc*_ (Figure 9A), although it was only about one-third of that induced by serosal addition (Figure 9B). To verify whether the action of luminal CCh is through the muscarinic receptors, atropine, a muscarinic receptor antagonist, was applied. As shown in Figures 9C,D, atropine (10 μM) added in mucosal side markedly attenuated luminal CCh-induced *I*_*sc*_ indicating that luminal CCh evokes duodenal *I*_*sc*_ via specific activation of muscarinic receptors expressed on the mucosal side of epithelial cells as well.

To test the extracellular Ca^2+^ effect in CCh-induced duodenal *I*_*sc*_, we omitted extracellular Ca^2+^ in each side of the tissues. Figures 9E,F show that luminal CCh-evoked *I*_*sc*_ was markedly suppressed when Ca^2+^ has omitted on the serosal side but not the mucosal side. Furthermore, we verified further that nifedipine (10 μM), an L-type calcium channel blocker, did not inhibit luminal CCh-induced *I*_*sc*_ either (Figure 9G). Together, these findings indicate that even luminal CCh induced Ca^2+^-dependent duodenal *I*_*sc*_ through serosal Ca^2+^ entry, further supporting our previous notion that CCh induced Ca^2^-dependent duodenal *I*_*sc*_ by serosal SOCE mechanism exclusively (Yang et al., 2018).

### Luminal Carbachol Induced Ca^2+^-Dependent Duodenal *I*_*sc*_ via Transient Receptor Potential Vanilloid 4-Constituted Store-Operated Ca^2+^ Entry

**FIGURE 10:**
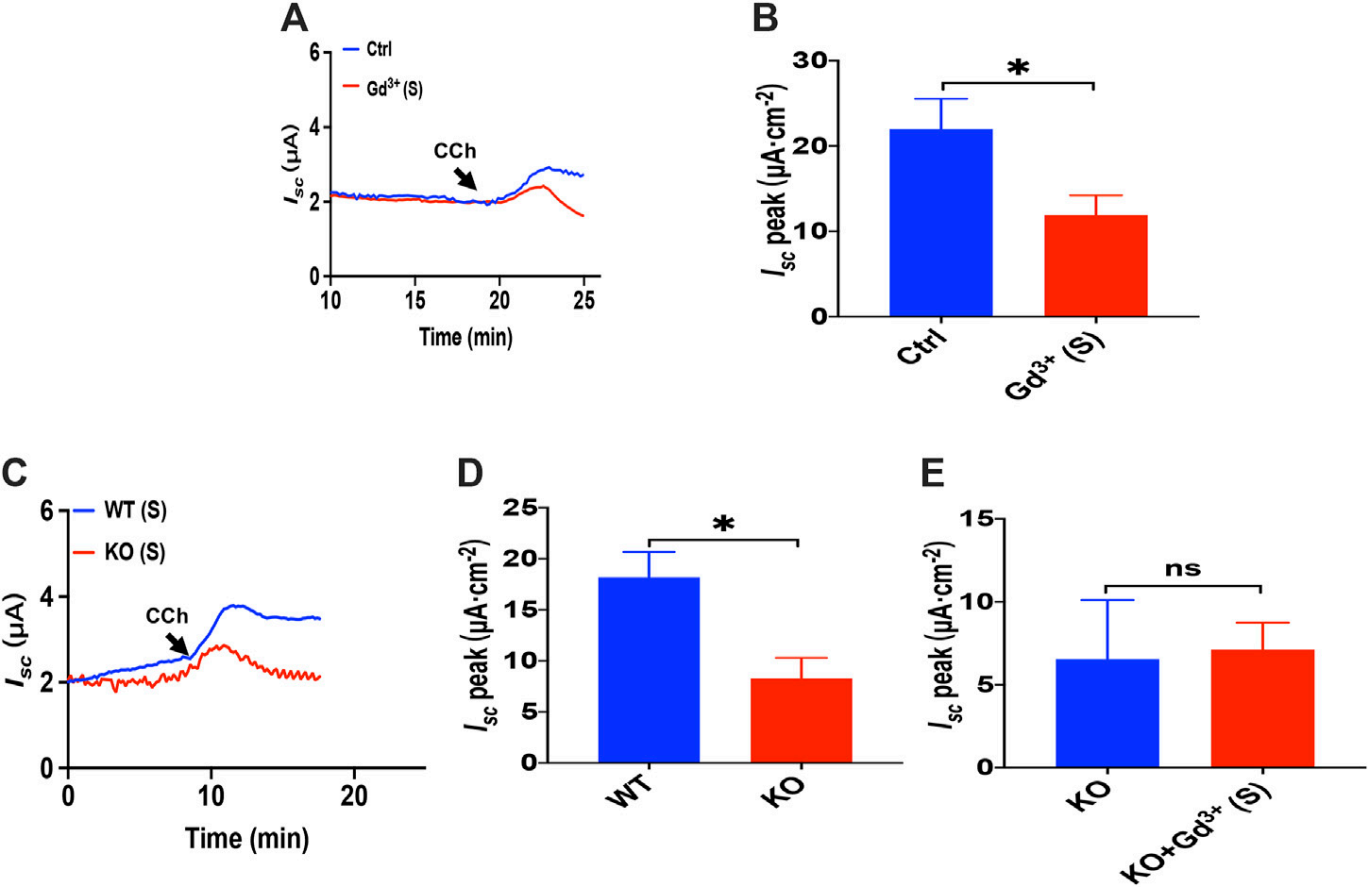
Luminal CCh induced Ca^2+^-dependent duodenal *I*_*sc*_ via TRPV4-constituted SOCE. **(A,B)** luminal CCh(100 μM)-evoked *I*_*sc*_ after GdCl_3_ (30 μM, *n* = 6) added to the serosal (s) side. **(C,D)** luminal CCh (100 μM) -evoked *I*_*sc*_ in wild-type (W.T.) or TRPV4 K.O. mice (*n* = 6). **(E)** luminal-stimulated *I*_*sc*_ after GdCl (Gd^3+^, 30 μM, *n* = 6) was added to the serosal (s) side of TRPV4 KO mice duodenum. Ctrl represents the control without drug treatment. Results are presented as mean ± SE. NS, no significant differences, *p < 0.05 vs. corresponding control by Student’s unpaired, two-tailed t-test.

As Gd^3+^ is a potential blocker of SOCE and CRAC/Orai channel, we added GdCl_3_ (30 μM) in the serosal side to test if it affects the luminal CCh induced Ca^2+^-dependent duodenal *I*_*sc*_ *.* As shown in Figures 10A,B, Gd^3+^ significantly reduced luminal CCh-stimulated duodenal *I*_*sc*_, which suggested luminal CCh evokes anion secretion through SOCE mechanisms.

To verify that TRPV4 is the molecular constituent of CRAC channels in the duodenum, we compared PGE_2_-stimulated duodenal *I*_*sc*_ between wild-type and TRPV4 KO mice. As shown in Figures 10C,D, duodenal *I*_*sc*_ induced by serosal addition of luminal CCh was significantly attenuated in TRPV4 KO mice. Meanwhile, GdCl_3_ (30 μM) serosal addition did not affect luminal CCh-stimulated duodenal *I*_*sc*_ of TRPV4 knockout mice (Figure 10E), further indicating that luminal CCh induced Ca^2+^-dependent duodenal anion secretion via TRPV4-constituted SOCE.

### Activator of Store-Operated Ca^2+^ Entry in the Duodenal Epithelium and TRPV4-Constituted Store-Operated Ca^2+^ Entry Mechanism in IECs

**FIGURE 11:**
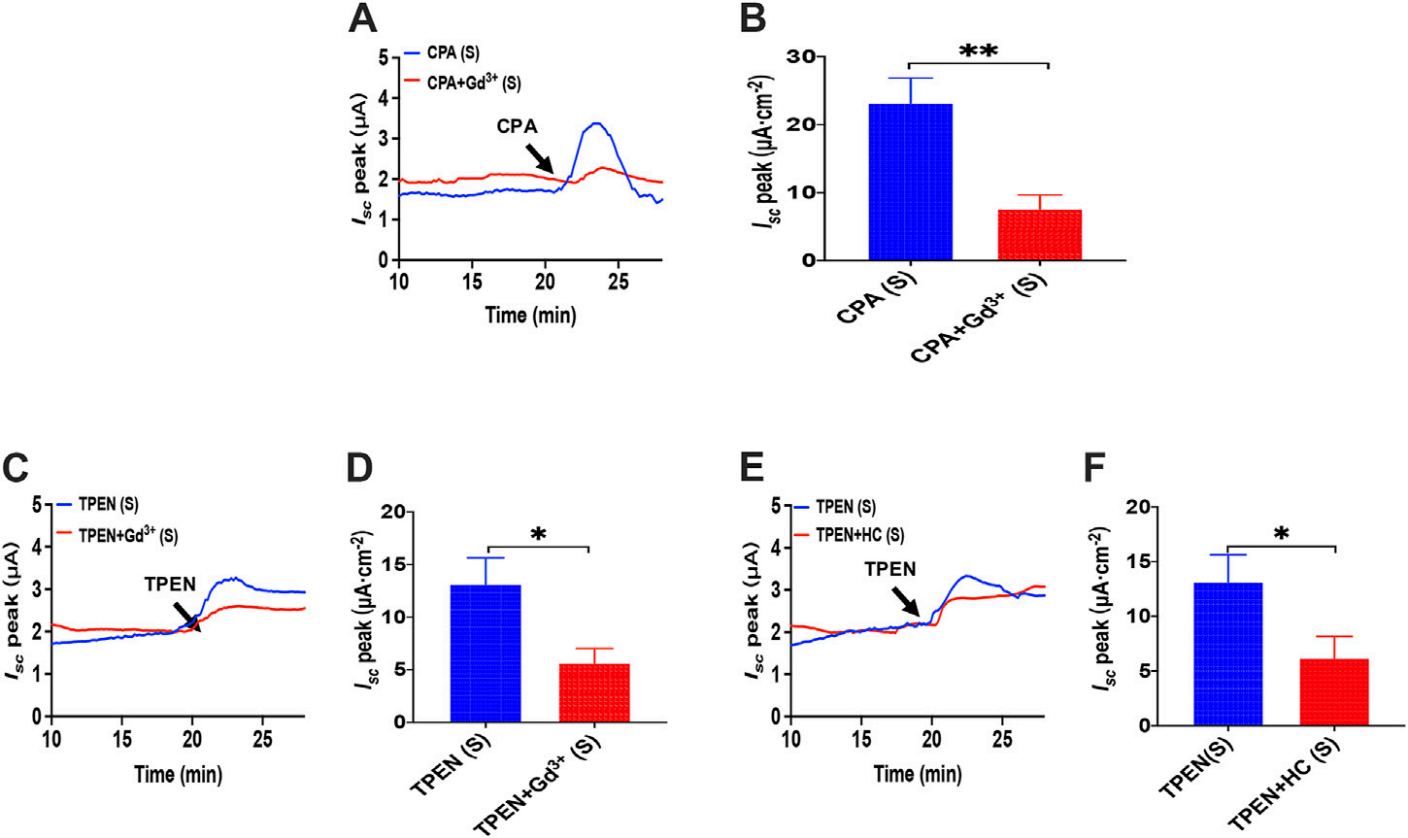
Activator of SOCE in the duodenal epithelium. **(A,B)** serosal CPA(10 μM)-stimulated duodenal *I*_*sc*_ after addition of GdCl_3_ (30 μM) in the serosal side (*n* = 6). **(C,D)** TPEN-stimulated duodenal *I*_*sc*_ after addition of GdCl3 (30 μM) in the serosal side (*n* = 6). **(E,F)** TPEN-stimulated duodenal *I*_*sc*_ after adding HC067047 (30 μM) to the serosal side (*n* = 6). Ctrl represents the control without drug treatment. Results are presented as mean ± SE. NS, no significant differences, *p < 0.05, **p < 0.01 vs. corresponding control by Student’s unpaired, two-tailed t-test.

As we have already known that cyclopiazonic acid (CPA), an ER-Ca^2+^-ATPase inhibitor (Dolmetsch and Lewis, 1994), can activate SOCE (Bird et al., 2008), TPEN also evoked SOCE by chelating Ca^2+^ within E.R. (Gwozdz et al., 2012). The addition of CPA (10 μM) in the serosal induced a transient high *I*_*sc*_ peak with a sustained phase following (Figure 11A), and the addition of GdCl_3_ (30 μM) in the serosal side significantly reduced CPA-stimulated duodenal *I*_*sc*_ (Figures 11A,B). The addition of TPEN (1 mM) in the serosal induced a transient high *I*_*sc*_ peak with a sustained phase following (Figures 11C,E), while the addition of GdCl_3_ (30 μM) and HC067047 (30 μM) in the serosal side significantly reduced TPEN-stimulated duodenal *I*_*sc*_ (Figures 11C–F).

**FIGURE 12:**
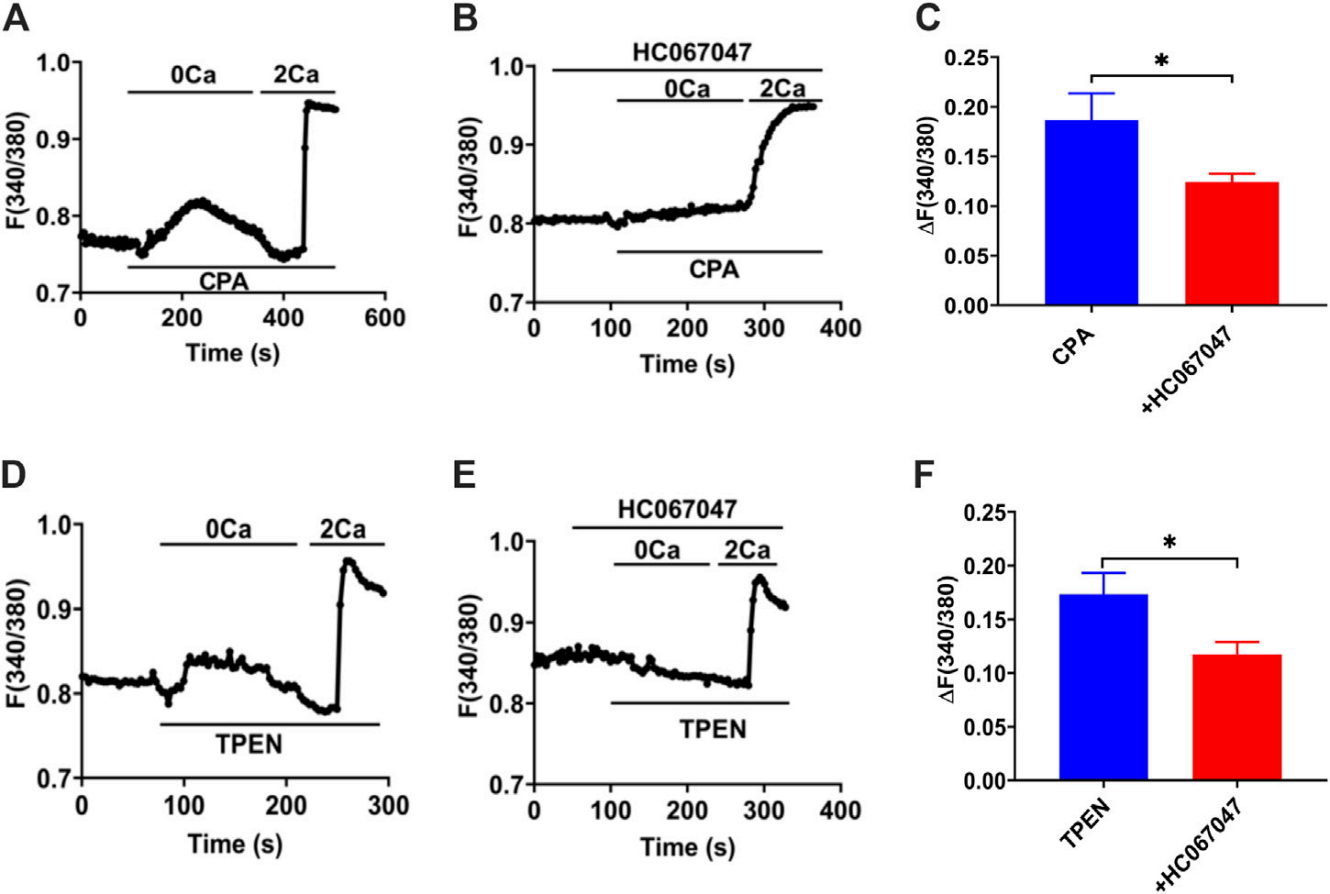
TRPV4-constituted SOCE mechanism in IECs. **(A)**Time courses of CPA (10 μM)-induced Ca^2+^ signaling in the extracellular Ca^2+^ omission (0 Ca, left) or not (2 Ca, right) in ICE-6 cells. **(B)** Time courses of CPA-evoked Ca^2+^ signaling with HC067047 (30 μM) pretreated in extracellular Ca^2+^ omission or not. **(C)** CPA-induced [Ca^2+^]_cyt_ mobilization with HC067047 (30 μM) pretreated in HEC-6 cells superfused with Ca^2+^ - containing solution (2 Ca). Results are presented as mean SE (*n* = 25–35 cells). **(D)** TPEN (50 μM) -evoked Ca^2+^ signaling in the extracellular Ca^2+^ omission (0 Ca, left) or not (2 Ca, right) in ICE-6 cells. **(E)** TPEN-evoked Ca^2+^ signaling with HC067047 (30 μM) pretreated in extracellular Ca^2+^ omission or not. **(F)** TPEN-induced [Ca^2+^]_cyt_ mobilization with HC067047 (30 μM) pretreated in HEC-6 cells superfused with Ca^2+^ - containing solution (2 Ca). Results are presented as mean SE (*n* = 25–35 cells) *p < 0.05 vs. corresponding control by Student’s unpaired, two-tailed t-test.

Our former studies, including T29 (Yang et al., 2018) and SCBN (Zhang et al., 2019; Zhang et al., 2021), confirmed that SOCE/ORAC mechanism is in IECs through SOCE blockers or knockdown cells. IEC-6 cells are usually applied as an IEC model to study intestine epithelial anion secretion (Wenzl et al., 1989), which has also been certificated the participation of SOCE mechanism (Chung et al., 2015). Meanwhile, TRPV4 expressed in IEC-6 cells basolateral and GSK1016790A induced [Ca^2+^]_cyt_ rising were suppressed by pretreatment with RN1734 or extracellular Ca^2+^ omission in IEC-6 cells (Yamawaki et al., 2014). So we measured [Ca^2+^]_cyt_ in IEC-6 cells to test the TRPV4 constituent SOCE mechanism. Superfused with Ca^2+^-free solution (0 Ca), CPA (10 μM), and TPEN (50 μM), first induced a rapid increase in [Ca^2+^]_cyt_ in IEC-6 cells as a result of ER Ca^2+^ release (Figures 12A,D). After completion of Ca^2+^ release from E.R., the restoration of extracellular Ca^2+^ (2 Ca) caused an enhanced increase in [Ca^2+^]_cyt_ owing to the SOCE mechanism (Figures 12A,D). As shown in Figures 12B,C,E,F, HC067047 (10 μM), a commonly used TRPV4 blocker in IEC-6, significantly inhibited CPA-and TPEN-induced SOCE, further advocating that TRPV4 participates in the composition of SOCE.

## Discussion

Albeit it is well recognized that the critical role of calcium signaling in epithelial ion transports of the salivary gland, pancreatic ducts, and colonic epithelia, the detailed regulatory in the small intestinal epithelial and the underlying molecular mechanisms are not fully understood. Our research demonstrates that: 1) serosal PGE_2_ stimulates anion secretion mainly through RyR/ER Ca^2+^ release-initiated serosal SOCE mechanism; however, serosal 5-HT and luminal CCh stimulate anion secretion mainly through IP_3_R/ER Ca^2+^ release-initiated serosal SOCE mechanisms. 2) CARC may act as the SOCE mechanism in the process of Ca^2+^-dependent anion secretion. 3) TRPV4 channels may represent the molecular constituents of serosal SOCE/CARC channels to mediate Ca^2+^-dependent anion secretion. Therefore, our results indicate that Ca^2+^ signaling is essential for three most common and important secretagogues-induced small intestinal anion secretion, in which serosal TRPV4-constituted SOCE mechanism may play a critical role. Thus, our findings supple a novel insight into the molecular mechanisms of secretagogues-mediated epithelial anion secretion via Ca^2+^ signaling.

Being a prevalent second messenger, [Ca^2+^]_cyt_ serve to regulate numerous cellular functions in various mammalian cells (Berridge et al., 2003), and it has been an essential regulator for intestinal epithelial ion secretion (Chew et al., 1998; Flemström and Isenberg, 2001; Jung and Lee, 2014). However, compared with excitable cells in which Ca^2+^ entry is mainly through VGCC, less is known about Ca^2+^ entry in non-excitable intestinal epithelial cells since functional VGCC may not be expressed (Parekh and Penner, 1997). In our research, we firstly confirmed the essential role of pure Ca^2+^ signaling in three secretagogues-induced small intestine anion secretion by using native mice duodenal epithelium, and then we revealed that they induced Ca^2+^ entry from the serosal side rather than from the mucosal side, which is consistent with our previous study (Yang et al., 2018). This phenomenon may be due to the following facts: 1) most secretagogues are derived from enterochromaffin (E.C.) cells or enteric neurons and transferred from the bloodstream, 2) their corresponding receptors are predominately located on the serosal side of intestinal epithelium, and 3) external Ca^2+^ concentrations in interstitial fluid of epithelia are maintained relatively consistent under physiological conditions.

It is well known that muscarinic receptors predominately express on the serosal side of the intestinal epithelium. However, we revealed for the first time that luminal CCh also evoked a significant duodenal *I*_*sc*_, which was inhibited by mucosal application of muscarinic receptor antagonist and omission of serosal calcium, suggesting that luminal CCh also activates mucosal muscarinic receptor to media Ca^2+^ entry from serosal side instead of mucosal side of duodenal epithelium. These new findings are not only mostly consistent with those obtained from our previous study but extend them (Yang et al., 2018). Although the muscarinic receptors in pancreatic acinar cells are localized to the apical side (Ashby et al., 2003), they are still elusive for their localization on the apical side of duodenal epithelial cells and their physiological significance. It was previously reported that bile acids might interact with apical muscarinic receptors on gastric chief cells and intestinal cells (Raufman et al., 2003); however, the detailed localization and significance of apical muscarinic receptors in the G.I. tract need further investigation.

Previous studies demonstrated that the serosal addition of CCh triggers IP_3_/IP_3_R/ER Ca^2+^ release that stimulates serosal SOCE mechanisms and ultimately induces Ca^2+^-dependent duodenal anion secretion (Yang et al., 2018; Zhang et al., 2019). Here, we further examined if PGE_2_ and 5-HT induced intestinal anion secretion through serosal SOCE mechanism. First, either omission of extracellular Ca^2+^ or ER Ca^2+^ chelation markedly attenuated PGE_2_- and 5-HT-induced duodenal *I*_*sc*_. Second, relatively selective SOCE blockers with different chemical structures significantly inhibited PGE_2_- and 5-HT-induced duodenal *I*_*sc*_ from serosal side instead of mucosal side of the duodenum. Third, GSK-7975A and Gd^3+^, selective *I*_CRAC_ blockers (Bird et al., 2008; Derler et al., 2013; Sataloff et al., 2017), also inhibited PGE_2_- and 5-HT-evoked *I*_*sc*_ from serosal side. Therefore, together with our previous studies, these findings support a universal role of serosal SOCE mechanism in Ca^2+^-dependent duodenal anion secretion induced by three most common secretagogues.

Furthermore, we reveal a difference between them: 1) dantrolene, a selective RyR antagonist, inhibited PGE_2_-induced but not 5-HT-induced duodenal *I*_*sc*_; 2) LiCl, an inhibitor of IP_3_ production attenuated 5-HT-induced but not PGE_2_-induced *I*_*sc*_. These findings suggest that ER Ca^2+^ release may be induced via different pathways: PGE_2_ and 5-HT activate RyR and IP_3_R, respectively. Although ER Ca^2+^ release is mainly mediated by two well-known channels RyR and IP_3_R, they have different Ca^2+^ affinities (Carreras-Sureda et al., 2018). 5-HT couples to IP_3_/IP_3_R signaling pathway in glioma cells, which subsequently cause intracellular Ca^2+^ release (Noda et al., 2003). In contrast, PGE_2_ induces Ca^2+^ release through RyR in bovine adrenal medullary cells (Shibuya et al., 2002). The concentration of dantrolene (300 μM) in our research is relatively high. However, it is consistent with our previous study (Zhang et al., 2019) that serosal application of dantrolene (100 μM) caused significant inhibition of caffeine-induced *I*_*sc*_, and an increase in the concentration of dantrolene (300 μM and 1 mM) dose-dependently enhanced the inhibition. However, whether the high concentration of dantrolene has a non-specific effect needs further investigation in RyR KO mice or cell lines. Moreover, 2-APB is a non-specific IP3R inhibitor, so we chose to examine the involvement of IP_3_R by inhibition of IP_3_ with LiCl. Further experiments in K.O. mice or cell lines may be needed to verify IP3R involvement in Isc and SOCE further.

Molecular components of SOCE in intestinal epithelial cells have not been well identified. They are considered as TRPC1 in IEC (Rao et al., 2006) or STIM1/Orai1 in rat colonic epithelium (Onodera et al., 2013) and Caco2 cells (Yang et al., 2018). TRPV4 channels are nonselective cation channels with higher permeability for Ca2+ (Gees et al., 2010) and expressed on the G.I. tract (Balemans et al., 2017). It was previously reported that SOCE could be constituted by TRPV4 alone or together with TRPC1 to form a heteromeric channel (Ma et al., 2011). Therefore, using TRPV4 antagonist and TRPV4-KO mice, we examined if it represents molecular components of SOCE in mouse native duodenum. Indeed, we demonstrate that TRPV4 channels may constitute SOCE to contribute to PGE_2_- and 5-HT-induced Ca^2+^-dependent duodenal anion secretion for the first time. In the meantime, we adopted a TRPV4 blocker to suppress SOCE activation in the IEC-6 cell line, which elucidates that TRPV4 participates in SOCE. However, GSK1016790A alone had no effect on the basal Isc in W.T., and K.O. mice, which could be due to the fact that directly opening TRPV4 channel itself may not be sufficient to cause calcium influx *in situ* because TRPV4 is only one of many components for SOCE activation, including STIM1, Orai, TRPC channels.

Although the role of TRPV4 channels in G.I. disease has been broadly examined (Vergnolle, 2014), their physiological roles in the gut are still elusive, which need further investigation.

**FIGURE 13:**
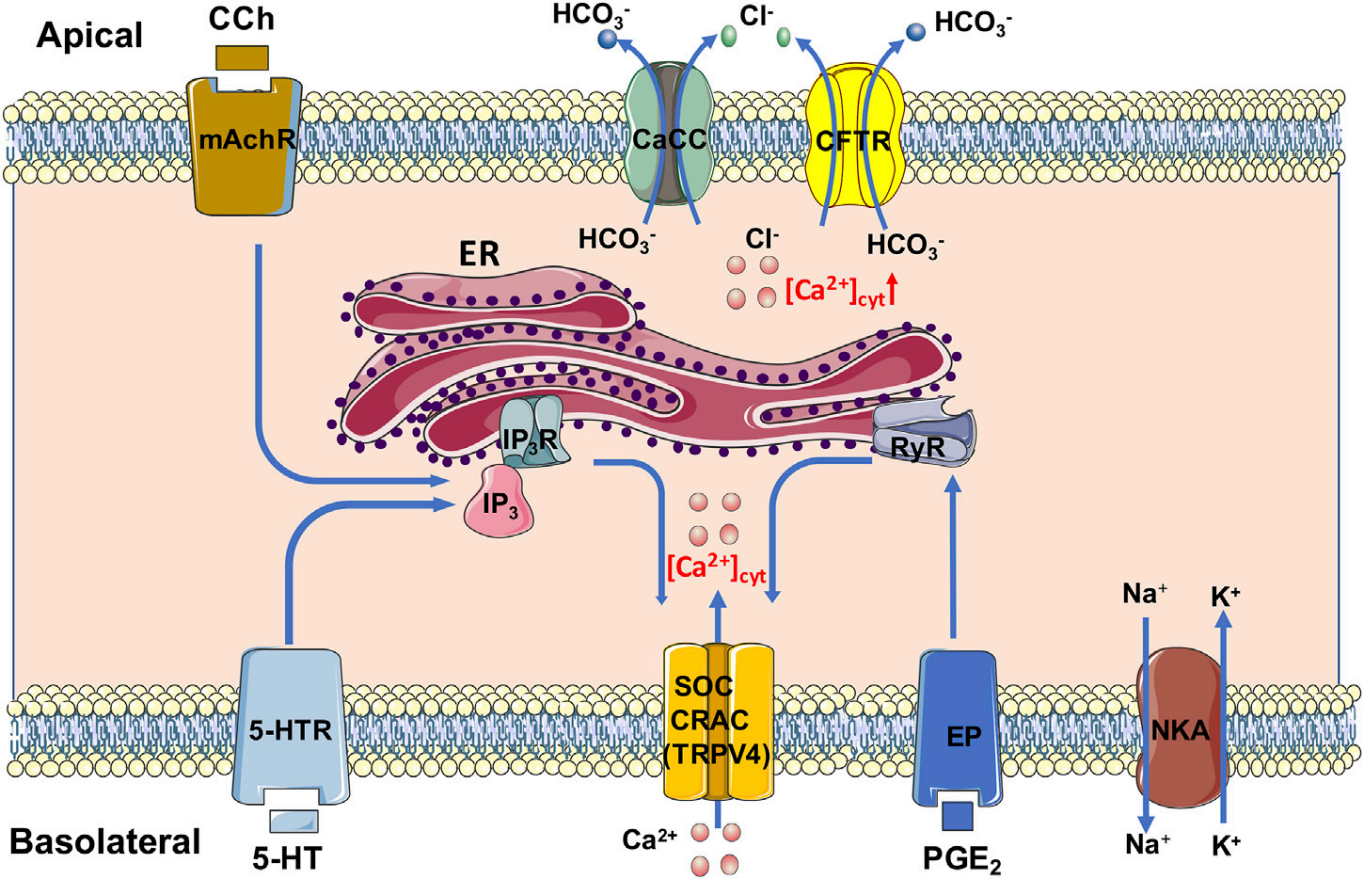
Mechanism diagram of secretagogues mediating Ca^2+^ signaling in duodenal epithelial cells via TRPV4-constituted serosal SOCE mechanism. PGE_2_ and 5-HT stimulate this mechanism exclusively from serosal side of the duodenum; CCh stimulates it from both sides. 5-HT and CCh trigger the ER Ca^2+^ release to initiate the SOCE likely via IP_3_/IP_3_R, but PGE_2_ triggers it likely via RyR. CaCC, Ca^2+^-activated Cl^−^ channels; CFTR, cystic fibrosis transmembrane conductance regulator; CRAC, Ca^2+^ release-activated Ca^2+^ channel; ER, endoplasmic reticulum; EP, PGE_2_ receptor; IP_3_ and IP_3_R, inositol 1,4,5-triphosphate and its receptor; mAchR, muscarinic acetylcholine receptor; NKA, Na^+^/K^+^ ATPase; RyR, ryanodine receptor; SOC, store-operated channels; TRPV4, transient receptor potential V4; 5-HTR, 5-HT receptor.

In conclusion, we underscore an essential role of Ca^2+^ signaling mediated anion secretion by TRPV4-constituted serosal SOCE mechanisms, which is universal for the three most common and important secretagogues. Although PGE_2_ and 5-HT stimulate this mechanism exclusively from serosal side of the duodenum, CCh stimulates it from both sides. We also reveal that 5-HT and CCh trigger ER Ca^2+^ release to initiate SOCE likely via IP_3_/IP_3_R, but PGE_2_ triggers it likely via RyR. A diagram is depicting, which we find in Figure 13. Complete comprehension of molecular mechanisms underlying secretagogues-mediated intestinal ion transports via Ca^2+^ signaling will enhance our knowledge of G.I. epithelial physiology and G.I. disease associated with abnormal anion secretion, such as diarrhea and constipation.

## Data Availability

The raw data supporting the conclusion of this article will be made available by the authors, without undue reservation.

## Supplementary Material

Supplementary Figure 1
**(A)** Time courses of GSK1016790A (10 μM)-induced *I*_*sc*_ in wild-type (W.T.) and TRPV4 K.O. mice (*n* = 6), while CCh verifies that the mice tissue is alive. **(B)** PGE_2_-evoked *I*_*sc*_ after HC067047 (30 μM, *n* = 6) was added to the serosal (s) side of the TRPV4 knockout (K.O.) mice duodenum. **(C)** 5-HT-evoked *I*_*sc*_ after HC067047 (30 μM, *n* = 6) was added to the serosal (s) side of the TRPV4 knockout (K.O.) mice duodenum. Ctrl represents the control without drug treatment. Results are presented as mean ± SE. NS, no significant differences vs. corresponding control by Student’s unpaired, two-tailed t-test.
