# Supplementary figures and images for: Role of Serosal TRPV4-Constituted SOCE Mechanism in Secretagogues-Stimulated Intestinal Epithelial Anion Secretion
Source: Front Pharmacol. 2021 Jul 14;12:684538. doi: 10.3389/fphar.2021.684538 (PMC8317263; doi:10.3389/fphar.2021.684538)

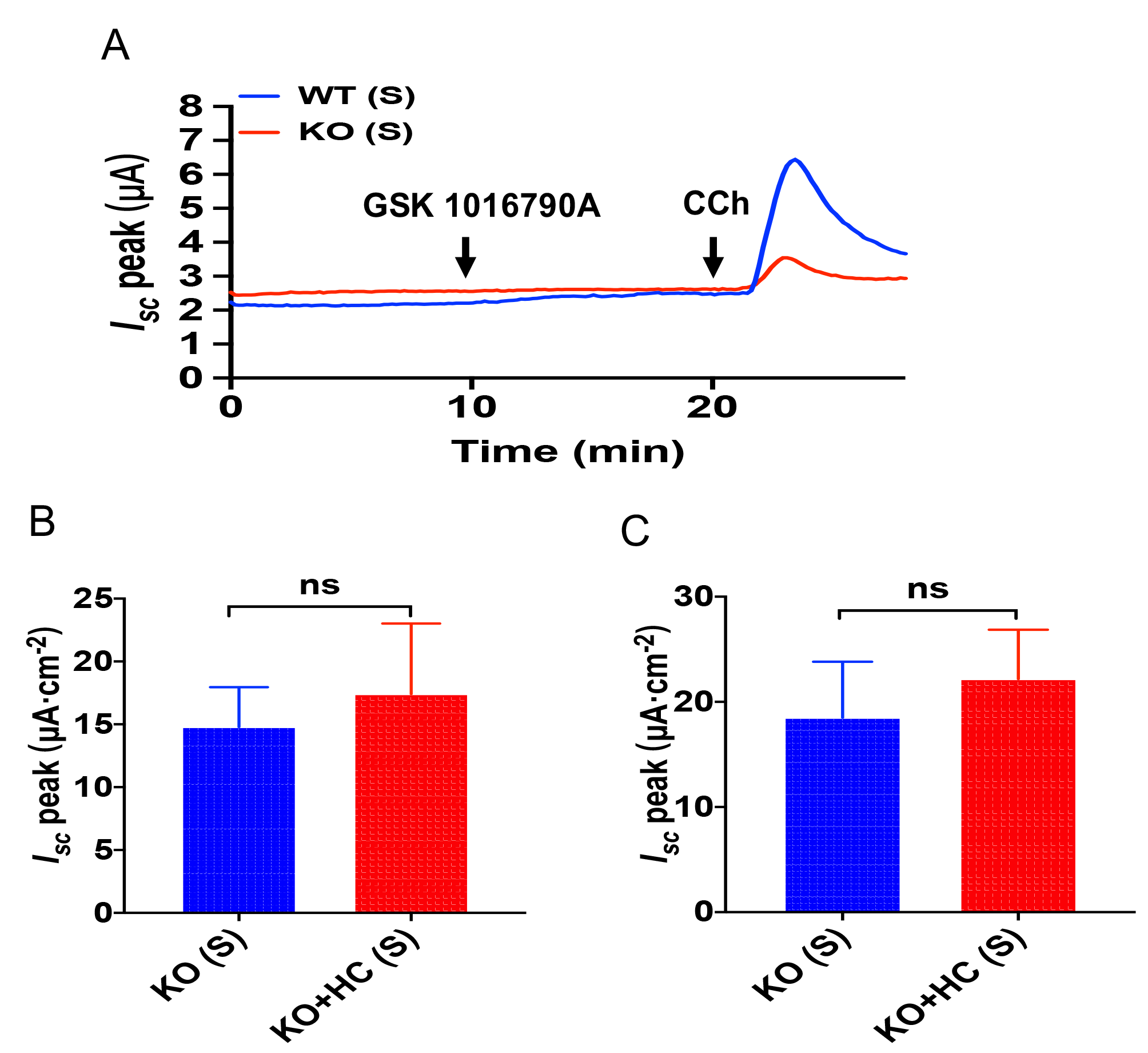

Supplement: Supplementary file 1 [file Image1.TIF]
